# 

**Published:** 1892-03-05

**Authors:** 


84, Vol. XI.—March 5th, 1892.

[...ered at the General Post Office as a Newspaper for ...mission in the United Kingdom and Abroad.]

WITH EXTRA NURSING SUPPLEMENT.

Per Annum, 6s. 6d., post free.

Monthly Parts, 6d.; post free, 8d.

Ditto per Annum, 8s., post free.

### THE HOSPITAL

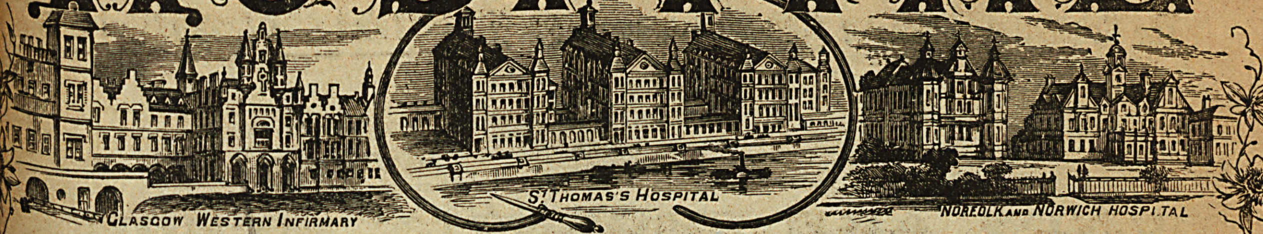

WITH EXTRA NURSING SUPPLEMENT.

No. 284. Vol. XI.] SATURDAY, MARCH 5th, 1892. [ONE PENNY.

